# The cystic fibrosis transmembrane conductance regulator (CFTR) and its stability

**DOI:** 10.1007/s00018-016-2386-8

**Published:** 2016-10-12

**Authors:** Xin Meng, Jack Clews, Vasileios Kargas, Xiaomeng Wang, Robert C. Ford

**Affiliations:** Faculty of Life Sciences, The University of Manchester, Oxford Rd, Manchester, M13 9PL UK

**Keywords:** CFTR, Membrane protein stability, Cystic fibrosis, Membrane protein structure, Biological detergent

## Abstract

The cystic fibrosis transmembrane conductance regulator (CFTR) is responsible for the disease cystic fibrosis (CF). It is a membrane protein belonging to the ABC transporter family functioning as a chloride/anion channel in epithelial cells around the body. There are over 1500 mutations that have been characterised as CF-causing; the most common of these, accounting for ~70 % of CF cases, is the deletion of a phenylalanine at position 508. This leads to instability of the nascent protein and the modified structure is recognised and then degraded by the ER quality control mechanism. However, even pharmacologically ‘rescued’ F508del CFTR displays instability at the cell’s surface, losing its channel function rapidly and it is rapidly removed from the plasma membrane for lysosomal degradation. This review will, therefore, explore the link between stability and structure/function relationships of membrane proteins and CFTR in particular and how approaches to study CFTR structure depend on its stability. We will also review the application of a fluorescence labelling method for the assessment of the thermostability and the tertiary structure of CFTR.

## Introduction

**Fig. 1:**
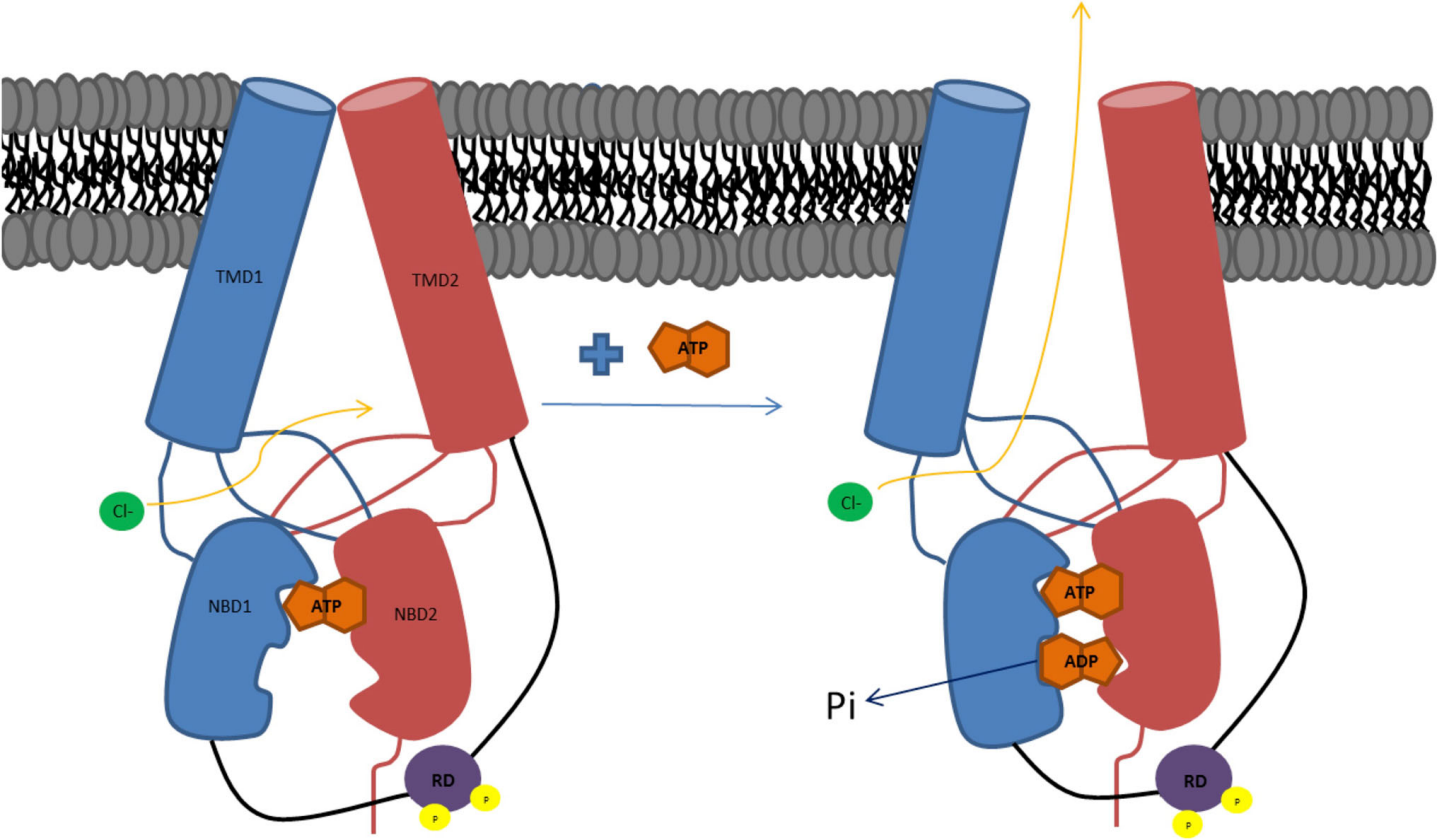
Cartoon illustrating how ATP binding and subsequent hydrolysis could lead to channel opening and flux of chloride ions (based on the model proposed by Wang et al. [3])

Cystic fibrosis transmembrane conductance regulator (CFTR) is unique among ABC transporters as it is the only member of this family of membrane proteins to act as an ion channel (although two ABC proteins (SUR1 and SUR2) regulate a potassium channel rather than act as transporters [1, 2]). As expected, the cystic fibrosis transmembrane conductance regulator (CFTR) follows the same domain structure as other ABC transporters (Fig. 1): it has two nucleotide-binding domains (NBDs) in tandem with two transmembrane domains (TMDs). What differentiates it from the other transporters (apart from its channel activity) is its regulatory domain or ‘R’ region (the word ‘region’ is suggested by Forman-Kay and co-workers as this 200-amino acid-long region is largely unstructured when analysed in isolation by NMR [3]). The R-region lies between the first TMD and the second NBD, within the cytoplasm. There is no significant sequence homology between the R-region and any other proteins in nature; hence, its origins are obscure. Within the ABC family, only ABCA1 in higher plants has a connecting region of similar length, and this is enriched in charged residues, like the CFTR R-region.

The R-region contains a number of Protein Kinase A (PKA) phosphorylation sites which have been shown to be highly conserved across species [4] PKA is a cAMP-activated kinase and as cellular levels of cAMP increase, PKA phosphorylates CFTR. In a readily understood model, the R-region is proposed to block the NBDs from associating together which subsequently keeps the channel in a closed conformation. Phosphorylation causes a structural change that removes the R-region from its steric-interfering position and allows NBD dimerisation to occur, triggering a much larger conformational change (Fig. 1). It is then the binding of ATP that is thought to initiate channel opening whilst channel closure is associated with ATP hydrolysis and release of ADP and inorganic phosphate [5, 6]. Forman-Kay and co-workers have proposed a more complex model involving the C-terminal region [7], which also displays a sequence unique to CFTR. In this model, phosphorylation of the R-region causes a switch from it interacting with the NBDs to it interacting with the C-terminal region. In this scenario the R-region acts as a global regulator of CFTR via its alternative interacting partners [7].

## The effects on stability of F508 deletion

Cystic fibrosis is caused by mutation in the CFTR protein. There are estimated to be over 1800 different mutations that can lead to cystic fibrosis (http://www.genet.sickkids.on.ca). Of these, the deletion of phenylalanine at position 508 is by far the most common and 95 % of CF patients possess at least one allele with this mutation. Homozygous F508del patients account for 65–70 % of cystic fibrosis cases in some groupings [8] and they have almost complete loss of the protein at the apical membrane. These patients have the most severe form of the disease. Heterozygous individuals (carriers), with one WT gene and one F508del mutated gene, have no symptoms and there may have been some selective advantage in the past for such individuals—such as reduced water loss in diarrhea. This has led to a theory that the prevalence of this mutation in certain populations is due to the increased resistance to dehydration-causing diseases such as cholera and typhoid fever which were previously major causes of morbidity, especially in infancy [9].

The F508del mutation and its effect on the stability and function of the protein have been studied extensively [10–14]. The deletion of phenylalanine at position 508 is crucial due to the position of this residue in the tertiary structure of the protein. It probably lies at the connecting position between the helical sub-domain of NBD1 and the second cytoplasmic loop of TMD2, a conclusion based on homology modelling [15] as well as cross-linking studies [8, 16, 17]. As a result of the mutation, there is thought to be a less stable conformation between TMD2 and NBD1, resulting in a protein that is recognisable by ER chaperones and which is then degraded by the ER quality control system via ubiquitinylation and proteasome-mediated degradation [18–20].

Cui et al. also considered the defect of F508del in terms of a different set of domain interactions [21]. They proposed that F508 deletion has an effect early in the folding of the protein and that the interaction between NBD1 and TMD1 is stabilised by this residue. These conclusions were based on the observation of a defined TMD1-NBD1 unit that was resistant to exogenously added protease in the WT protein, but not in the F508del version. This early unit in the CFTR folding pathway was proposed to be sufficient to escape endoplasmic reticulum quality control. If the F508del CFTR protein arrives at the apical membrane of the epithelial cell, it exhibits a reduced functionality as a chloride channel [22] and is susceptible to degradation. Two problems are thus associated with the mutated protein, a reduced migration to the apical membrane of the cell and the limited amount of protein that is found at the membrane has a highly reduced gating functionality. Treatments aimed at combating both of these defects have been considered [14].

## Treatments affecting the stability defect

The first indication of a potential therapeutic method was reported by Denning et al. [23]. Their results showed, with cells from two different expression systems, that the induction of expression of F508del CFTR at lower growth temperatures gave a much greater amount of fully glycosylated protein (the so-called ‘Band C’, migrating with a roughly 25 kDa higher relative mass on SDS-PAGE gels is extensively glycosylated). They suggested that some of the mutated protein had escaped the usual endoplasmic reticulum quality control step and had been correctly glycosylated and had proceeded to the plasma membrane. Perhaps for the first time these results highlighted the role of thermal stability in the correct processing of full-length CFTR. Shortly after this, work by Sato et al. [24] showed that F508del-CFTR-expressing cells, when exposed to 10 % glycerol, increase their expression of the mutant protein. These results included immunoblotting of the mature, fully glycosylated protein after pulse chase and patch clamp recordings of the cells which identified an increase in chloride conductance. This discovery, alongside that of Denning and co-workers [23], illustrated that the instability/misfolding of F508del-CFTR can be reversibly corrected by either lowered thermal energetics of the protein, or by the inclusion of a molecular chaperone such as glycerol. Although glycerol treatment cannot be translated therapeutically, the work highlighted that development of small-molecule correctors of the instability/folding defect might achieve pharmacological practicality. It is now 20 years after these studies, and there has been the discovery of a number of pharmacological correctors aimed at treatment of the F508del mutation [25]. For example, compounds have been produced that affect the F508del-CFTR interactome including the proteasome complex that leads to degradation of the protein and chaperones that specifically bind to the F508del-CFTR peptide [13, 26, 27].

One of the earliest pharmacological groups of compounds to be discovered was that of the tricyclic benzo[c]quinoliziniums or MPB compounds [28]. These were originally deduced as activators of the CFTR channel. A further study of these compounds carried out by Dormer et al. [29] revealed that application of a derivative of the original compounds, MPB-07, resulted in increased delivery of CFTR to the apical membrane, similar to the amount seen in wild-type cells. This was shown to be as a result of the prevention of normal degradation of the mutated protein, allowing for increased, yet unstable CFTR to make its way to the apical membrane.

Some of the first pharmacological chaperones discovered through high-throughput drug screening were reported by Pedemonte et al. [30]. Four classes of molecules were found by this group of workers, each showing some efficacy in helping to re-fold CFTR. Out of the four classes of molecules, two showed significant efficacy in promoting an increased amount of F508del-CFTR to the plasma membrane. Those of the bisaminomethylbithiazoles, class 4, were found to be the most effective. Two particular molecules, corr-4a and corr-4c, when incubated with CFTR-expressing Fischer Rat Thyroid (FRT) cells, showed an increased efficiency of CFTR folding as well as stabilisation of the protein. Classes 2 and 4 are both thought to be proteostasis regulators [31], due to their effect on either the endoplasmic reticulum quality control (class 4) or the lysosome-mediated degradation quality control system (class 2). Only class 4 molecules showed efficacy in primary bronchial epithelial cells which would be the primary target cell in the diseased lungs.

Pedemonte and co-workers went on to explore the class 2 molecules identified in the original screening; this led to identification of a particular group of compounds called aminoariathiozoles (AATs). These compounds showed enhanced channel trafficking and improved gating; this was seen as a promising result due to the fact that a potentiator would no longer be required in addition to a corrector. However, a further study involving these compounds failed to produce a statistically significant result in primary bronchial epithelial cells [32]. A number of studies have similarly fallen short in finding molecules that are effective in vivo.

Van Goor et al. [33] described a corrector compound with efficacy in human bronchial epithelial cells, a quinazolinone derivative, VRT-325. From their study, they showed that the drug also aided the expression of other mutant forms of CFTR. Further optimisation from this screen led to one of the most successful correctors to be developed [34]. When tested in cultured primary human bronchial epithelial cells, VX-809 increased chloride transport to that of 14 % of non-CF (HBE). Most importantly, this was enough to produce an increase in the thickness of the airway surface liquid (ASL) which shows a high clinical importance [35].

VX-809 did show some efficacy in Phase IIa clinical trials carried out by Clancy et al. [36]. These studies show a reduced sweat chloride concentration indicating that VX-809 is active in that particular organ. However, reduced sweat chloride is only a biomarker; it does not improve quality of life. A desired clinical outcome in CF patients would be improved lung function. This particular study proved that VX-809 has an acceptable safety profile and provided a foundation for an increase of the study on the particular drug.

Farinha, CM et al. [37] have shown that out of the three correctors currently developed: VRT-325, VX-809 and C4, VX-809 has been shown to be the most effective in increasing protein accumulation to the plasma membrane. Compared to VRT-325 and C4 in primary HBE cells, a higher production of F508del-CFTR was found with the use of VX-809. However, despite the promising results gained from VX-809, the rescuing effect was not high enough to restore CFTR function to wild-type levels. Cystic fibrosis symptoms may be prevented with as little as 25 % delivery of CFTR to epithelial cells, and certainly heterozygous individuals with presumably 50 % CFTR levels are unaffected. Synergistic trials were undertaken to try to increase the rescuing effect of VX-809 by applying another drug in the presence of VX-809. VX-770 is an FDA authorised potentiator drug, used to correct the gating defect present in the CFTR G551D mutation. This restores the channel into an open state which allows chloride conduction across the membrane where previously the G551D mutation resulted in a constitutively closed formation. Two recent studies have explored the use of VX-770 in combination with VX-809 to potentially increase the efficacy of VX-809’s rescue of F508del-CFTR to the plasma membrane.

Sampson et al. [38] showed development of a pharmacological chaperone corrector; that is, a compound that binds to the CFTR protein itself and stabilises the interaction between NBD1 and the TMD1. Despite no further work being carried out on the compound to date, Sampson and co-workers’ study provides somewhat of a scaffold for future pharmacological chaperone development. In particular, they identify a terminal phenyl ring which they postulate to be necessary for interaction with the nucleotide-binding domain.

VX-661 is another promising pharmacological chaperone, an improved version of VX-809, which has shown improved lung function among homozygous F508del-CFTR patients in a phase II trial. Vertex Pharmaceuticals has announced the compound to be used in triple combination trials with newly developed correctors, VX-440 and VX-152, both of which are thought to have a positive effect on the processing and trafficking of the CFTR protein leading to a higher yield at the cell surface.

Despite the positive data achieved from Vertex’s compounds, VX-770 has been shown to reduce the therapeutic effect of established CFTR chaperones. Two studies by Veit et al. [39] and Cholon et al. [40] have shown evidence which would suggest that VX-770 biochemically destabilises F508del-CFTR, thus reversing the effect of current correctors, VX-809 and VX-661. Although these are both in vitro studies and do not contradict the slight alleviation of CF symptoms by VX-809-derived treatments, discovery of new pharmacological correctors is probably required to ensure more effective treatment of the F508del mutation. A revised strategy to ensure effective drug discovery is also required, involving methods which increase the stability of the nascent protein, allowing it to progress to the plasma membrane. A compound of this nature will then suitably work in combination with VX-770 or another potentiator to maintain an effective amount of CFTR-F508del at the plasma membrane.

As renewed efforts are made towards the development of pharmacological compounds, assays of protein stability will be important to determine the efficacy of corrector compounds. One could argue that the more stable the protein, the less likely it will be to unfold and the more likely it will be that it will escape the ER quality control steps. On the other hand, the global thermodynamic stability of the mutated protein may be irrelevant if the primary defect is at the early stages of protein folding. Although data on low-temperature rescue of F508del CFTR and its subsequent residence time at the plasma membrane point to the likelihood of a global thermodynamic defect, there remains the possibility that a specific step in the folding pathway is disrupted. To address this, studies on the stability and structure of purified CFTR are desirable, and this will be addressed in the next section.

## Membrane proteins and the importance of stability for expression, purification and structural studies

Membrane proteins compose a significant fraction of the number of different proteins in the cell, and represent a disproportionately large fraction of the molecular targets of current drugs used to treat human disease. However, membrane proteins are vastly under-represented in the databases of protein structures [41]. This deficit in information is due to difficulties in membrane protein expression, purification and structure determination.

Membrane protein expression is fundamentally limited by available membrane area in the cell and by the capacity of the co-translational membrane insertion machinery. As a result, overproduction of membrane proteins like CFTR where membrane capacity is exceeded can lead to the production of aggregates in the cell that are highly resistant to detergent solubilisation and purification [42–44].

*Purification* is limited by the requirement for solubilisation of the membranes with detergent and then the need for detergent to be present throughout the subsequent fractionation processes [45]. The detergent may only partially solubilise the protein (if a ‘mild’ detergent is used) or may solubilise effectively but at the same time denature the protein (if a ‘harsh’ detergent is used [46, 47].

If a non-denatured protein can be purified, structural studies are still limited by the need for long-term stability of the tertiary and/or quaternary structure in the detergent, especially for NMR studies where the experiment will be performed well above 4 °C to increase the tumbling rate [48]. Moreover, the presence of the detergent belt surrounding the protein can interfere with the structure determination. For NMR studies the micelle belt increases the size of the protein/detergent complex and reduces tumbling speed, with consequently broadened signals [48]. This has led to a bias in the range of detergents that have been used for NMR structures of membrane proteins (http://www.drorlist.com/nmr/MPNMR.html) with those forming small micelles being favoured. For X-ray crystallographic studies, the micelle must be accommodated within the crystal lattice without interfering with lattice contacts, hence must also be small and perhaps deformable. It is probably for this reason that membrane protein crystals are biased towards relatively high solvent contents—that is they have an open lattice that can accommodate the presence of the detergent micelle [49]. For single particle cryo-electron microscopy (cryo-EM) studies, the detergent micelle can be visualised as a weaker/diffuse band of density around the protein [50]. It is not yet clear whether this reduces the effectiveness of the cryo-EM approach, but the background presence of free detergent micelles can reduce contrast and the efficiency of single particle alignment steps in the 3D reconstruction procedure. For small-angle X-ray scattering studies (SAXS), the micelle’s scattering needs to be divorced from the protein scattering to generate reasonable models which require a series of painstaking experiments with different solvent compositions so that either the protein or the detergent scattering component can be contrast-matched to remove its contribution to the scattering curve [51]. Similar approaches are employed for neutron scattering studies, but here the contrast is adjusted by varying the D_2_O to H_2_O ratio [52].

*Stability* Many of these difficulties for membrane protein structure determination can be influenced by the stability of the protein. For example, a relatively unstable membrane protein such as CFTR may be recognised as such by the host cell’s quality control machinery and degraded [14]. Similarly, an unstable protein is likely to have any instability accentuated by the presence of a detergent micelle; indeed the detergent may affect the membrane protein’s soluble domains as well as the transmembrane domains [53]. Locally unstable regions of the protein in domains normally exposed to the aqueous milieu may give poor water solubility during purification because hydrophobic residues that are normally buried in the tertiary fold will become exposed with a concomitant chance of forming polydisperse aggregates, especially at high protein concentrations [45, 54, 55]. Monodispersity is a strict requirement for structure generation from NMR and SAXS experiments, and is usually a pre-requisite for obtaining 3D crystals that diffract X-rays to high resolution [45, 56]. For single particle cryo-EM, monodispersity is also desirable, but is less strictly required for structure determination, especially if polydisperse aggregates can be readily distinguished from the non-aggregated monomeric or oligomeric particle [57]. Single particle cryo-EM and, to a similar extent, SAXS do not require high protein solubility for structure determination as they are compatible with protein concentrations below 1 mg/mL. However, NMR and X-ray crystallography usually require the concentration of the protein to be greater than 5 mg/mL.

Thus, the effects of the stability of a protein on its water solubility as well as its monodispersity properties are crucial for most structural biology techniques, with the possible exception of cryo-EM which is more tolerant of some polydispersity and does not require high water solubility of the protein. However, cryo-EM and SAXS-derived structures have typically been at much lower resolution than those obtained by X-ray crystallography and NMR. This is likely to change in the next few years for the cryo-EM technique with the introduction of new detection devices and software [58, 59]. Finally, it is possible that a membrane protein may adopt a non-native state that may still crystallise and generate a structure [60]. Thus, detergents and stability may be a factor even where structures are present in the protein data bank.

## Measures of protein stability

For many membrane proteins, stability can be measured simply by detecting the activity of the protein as a function of time, temperature or denaturant concentration. For example, crystallisation of G protein-coupled receptor family members has been achieved by finding stabilising mutations, with stability assessed by measuring radioactive ligand binding as a function of temperature [61]. For proteins without an obvious activity read-out, or where activity measurements require excessive amounts of purified protein, alternative biophysical measures of stability are available. Biophysical methods such as small-angle X-ray scattering, differential scanning calorimetry and circular dichroism have been well described and are generally easily interpretable, but have the drawback of consuming significant amounts of purified protein. Fluorescence methods are particularly useful for membrane proteins because of sensitivity, avoiding the consumption of large amounts of protein. Intrinsic fluorescence, where the differential properties of buried versus water-exposed tryptophan residues can be exploited, has been employed with membrane proteins, but the approach is hampered by the presence of detergent micelles or liposomes (where the protein has been reconstituted) [62]. Adding fluorescent probes has also been employed to measure membrane protein stability, although again, the presence of detergents or lipid complicates the interpretation of the data. The efficacy of three of the most commonly employed fluorescent reporters with two different purified membrane proteins was recently compared [63], and this provides a useful assessment of the methodology as applied to membrane proteins. Of note was that the cysteine (Cys)-reactive reporter 7-diethylamino-3-(4′-maleimidylphenyl)-4-methylcoumarin (CPM) was capable of distinguishing the unfolding of separate subunits of cytochrome c oxidase. We review the use of this reporter for CFTR stability measurement later.

## Expression and purification of CFTR and the effects of stability

The expression and purification of CFTR has been reported previously [54, 64–67]. Challenges in its purification are manifold and it illustrates many of the problems described in the previous section. For CFTR, a comparison of constructs with different degrees of stability is informative and shows how this factor affects expression and purification. As discussed above, F508del CFTR is less stable than the WT protein, whilst the G551D CFTR mutation locks the channel in a closed state, and this mutant form of the protein appears to be more stable than the WT protein. Comparison of expression levels for these constructs can be made by monitoring a GFP tag on the C terminus of the CFTR protein. The degree of GFP fluorescence can be compared with fluorescence from an intrinsic fluorescent protein (e.g. yeast mitochondrial succinate dehydrogenase) to allow the CFTR expression level to be adjusted for cell density and to allow for variability in the harvesting and breakage of cells and for any and gel loading errors.

**Fig. 2:**
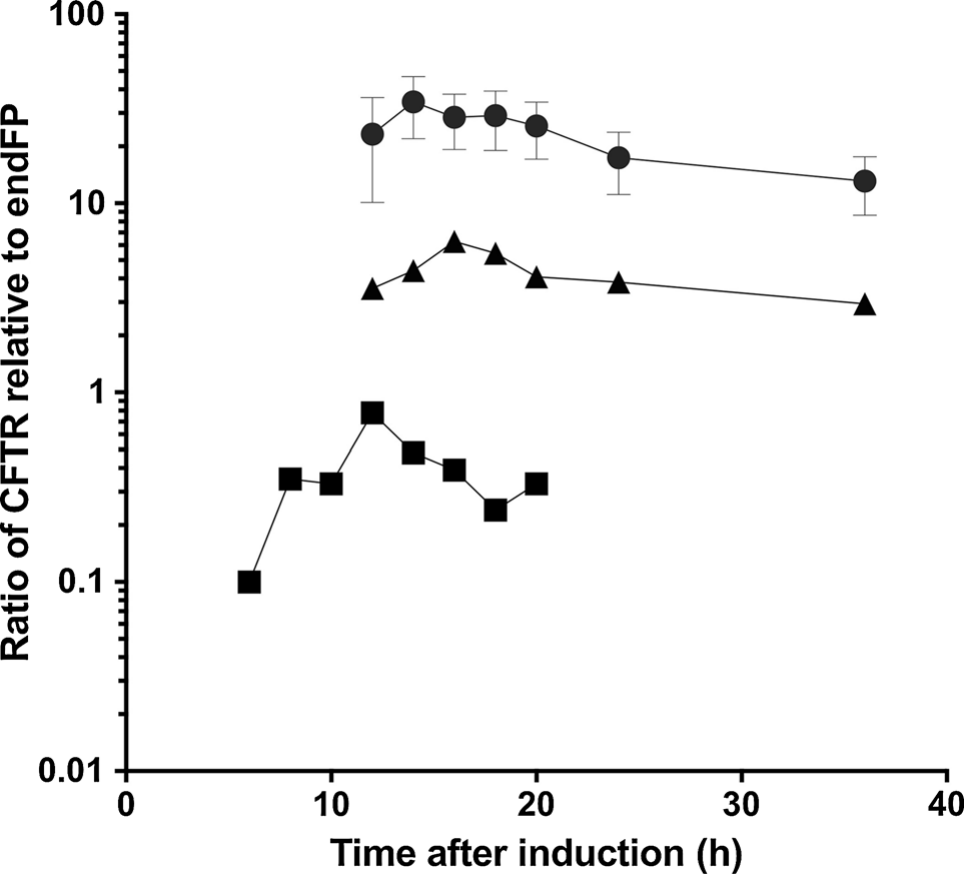
Expression levels are broadly related to stability for CFTR in *S. cerevisiae*. Comparison of WT (*triangles*), G551D (*circles*) and F508del (*squares*) CFTR expression as a function of time after induction. The fluorescence from the GFP tag attached to CFTR is compared to that from an endogenous fluorescent protein (endFP, succinate dehydrogenase) to correct for variability in cell breakage, microsome preparation and gel loading

An illustration of such data for the CFTR expression is shown in Fig. 2. As predicted there is a correlation between the stability of the construct and levels of protein detected in the same expression system. G551D CFTR had a much higher yield than WT or F508del CFTR. It is known that the potentiator VX-770 is able to restore channel activity to the G551D CFTR channel and to stimulate channel activity of WT and F508del CFTR; there is predicted to be a loss of some stability as a result [40]. A comparison of expression levels of G551D CFTR in cells treated with and without the potentiator VX-770 is shown in Fig. 2b. Cells treated chronically with VX-770 after induction did not show a reversal to the expression levels of WT CFTR.

## Purification of CFTR

CFTR is also an example for how stability can influence the ability to purify a protein, and detergents used to solubilise and purify CFTR can be compared. For example, we can examine the purification of the protein in a mild detergent and in a harsher anionic detergent:

### Purification of CFTR in an anionic lyso-lipid surfactant: lyso-phosphatidyl glycerol-14 (LPG14)

**Fig. 3:**
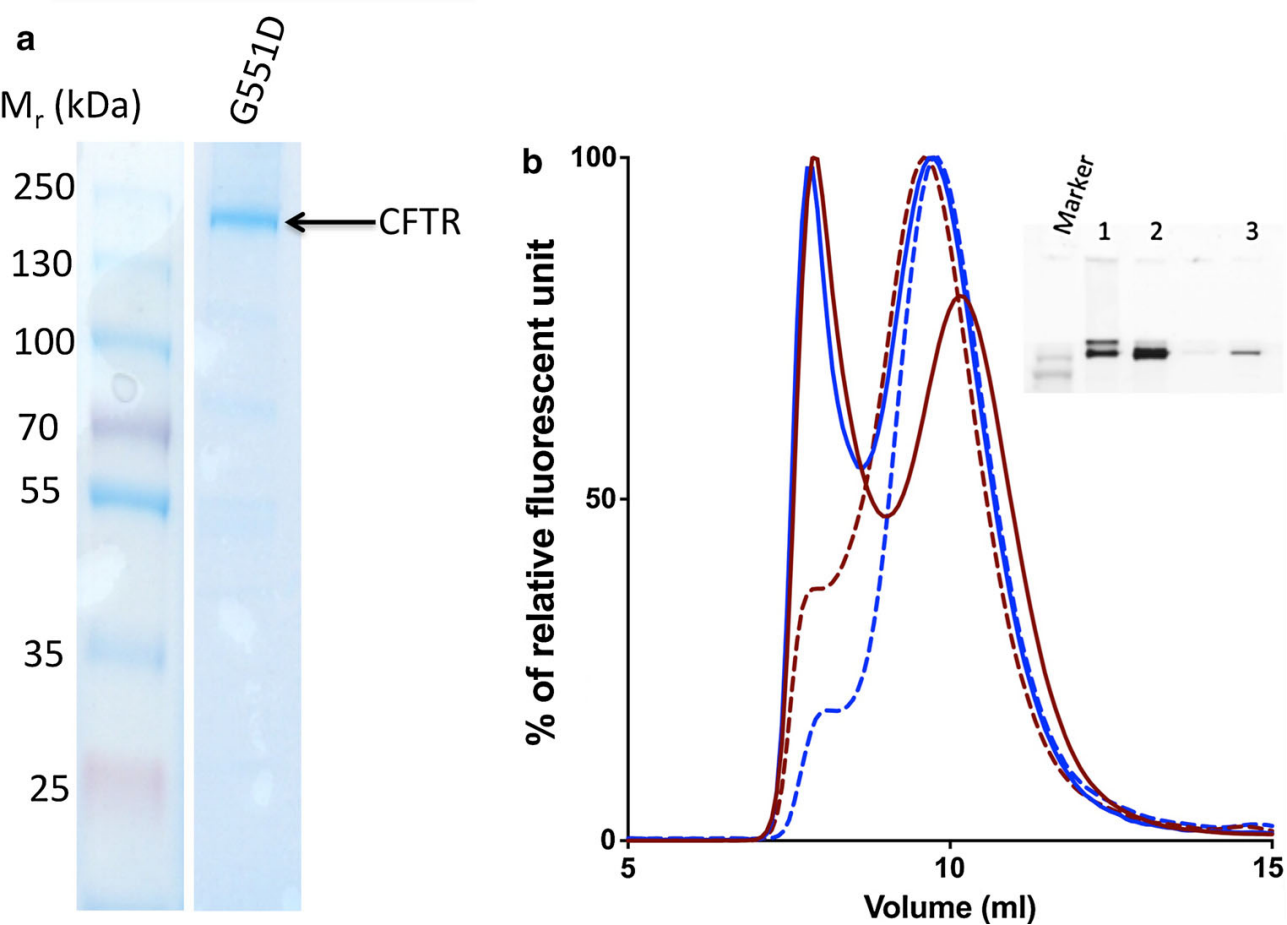
**a** Coomassie-stained SDS-PAGE gel of G551D CFTR eluted from the main peak from the nickel-NTA purification step for protein extracted and purified with LPG. **b** SEC purification of WT (*red line*) and G551D (*blue line*) CFTR in LPG. The second peak fractions (*solid lines*) were re-injected onto the Sucrose 6 *column* and the *curves* are shown on the same *graph*, WT (*red dashed line*) and G551D (*blue dashed line*). The inset is an example gel of the purification procedure. *Lanes 1* and *2* are the first and second fluorescence peak elution fractions. *Lane 3* was the re-injected fluorescence peak elution for G551D CFTR. The intervening track contains the tail of the first elution peak (monomeric CFTR in small amounts)

The anionic detergent, LPG is thought to be less mild in terms of its surface activity compared to non-ionic detergents [68]. In agreement with this, it was more efficient in solubilisation and purification. However, functional CFTR was successfully purified using this detergent [69, 70]. Figure 3 shows a Coomassie-stained SDS-PAGE gel of G551D CFTR purified using nickel-NTA affinity chromatography in 0.1 % LPG. Nearly 90 % of total CFTR in the microsomes can be solubilised in LPG and its binding efficiency to the nickel-NTA column can reach 90 % [67]. As a maximum, 20 mg of purified G551D protein can be obtained from a single 18-L fermenter run, compared to about 2 mg for WT CFTR [67]. These estimates of efficiency were estimated based on the GFP fluorescence of the fractions using a fluorometer. This tenfold increase in final yield of purified G551D protein compared to wild type [67] is in broad agreement with the cellular expression levels indicated in Fig. 2. The purity immediately after nickel-NTA affinity purification has been reported to reach 95 %, as judged by Coomassie-stained SDS-PAGE gels, but CFTR protein can then be injected onto a size-exclusion chromatography (SEC) column. This also allows monodispersity of the CFTR to be estimated as well as providing a polishing step for the purification. A fluorescence detector connected to the outlet from the SEC column allows the detection of either GFP fluorescence or tryptophan fluorescence. An illustration of a purification run is shown in Fig. 3.

**Fig. 4:**
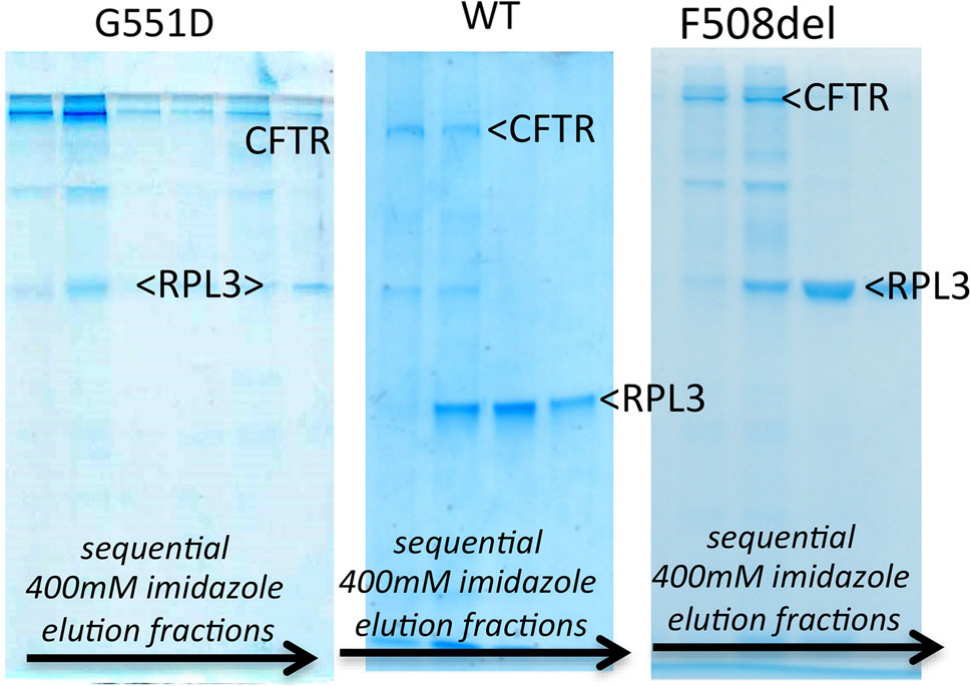
Coomassie-stained SDS-PAGE gels of (*left* to *right*) G551D, WT and F508del CFTR eluted in the 400 mM imidazole elution step from the nickel-NTA column for protein extracted and purified with DDM. The CFTR band (220 kDa) and the main contaminating protein in each case (RPL3: 44 kDa) are indicated. The intermediate contaminating band is an abundant yeast membrane protein (plasma membrane H^+^-ATPase:100 kDa)

Interestingly, the more stable G551D CFTR construct also seems to be more homogeneous than WT CFTR, as judged by the relative fluorescence of the larger aggregates versus monomer peak (Fig. 4). For both G551D and WT CFTR proteins it is possible to concentrate them using a 100 kDa MWCO membrane concentrator up to 30 mg/mL in the LPG detergent. In general, the yield of LPG-purified G551D CFTR is high compared to other eukaryotic membrane proteins with the same GFP tag and yeast expression system [71]. The homogeneity of the protein and high yield makes it possible to employ biophysical characterisation methods that demand large amounts of protein and also to attempt high-resolution structural studies. For example, both WT and F508del CFTR protein purified in LPG have been used in small-angle X-ray scattering studies and for Cryo-EM [51].

### Purification of CFTR in a non-ionic detergent: dodecyl maltoside

DDM is the most commonly used detergent to purify membrane proteins and it has been the most successful for crystallisation [72]. Purification methods in both DDM and LPG are similar, as described in [67]. Figure 4 shows a comparison of Coomassie-stained SDS-PAGE gels of CFTR eluted after nickel-NTA affinity purification in 0.1 % DDM. Example gels of G551D, WT and F508del CFTR (left to right) are given. The main contaminating protein in the CFTR purification is yeast ribosomal protein L3 (RPL3, as confirmed by mass spectrometry), which clearly binds strongly to the Ni–NTA resin, eluting later than CFTR in a 400 mM imidazole elution step. Compared to WT CFTR purification, the G551D purification again appears to be more efficient, with equivalent levels of RPL3 present, but considerably higher levels of the full-length CFTR. WT and F508del CFTR show similar levels of purity after Ni-NTA affinity purification. Yields of G551D CFTR after the nickel-NTA affinity purification with DDM were reported to be about 3–4 mg from an 18-L fermenter while the yield was typically lower (2–3 mg) for WT CFTR [67]. The differences between G551D and WT protein can also be noted at the early stages of purification, with more solubilisation of the initial microsomal G551D CFTR by DDM. G551D can be solubilised to about 50 % yield with DDM (occasionally above 80 % can be observed) whereas typically 30 % solubilisation efficiency can be gained for WT CFTR. Concentrating DDM-purified CFTR proteins after affinity purification is a problem. The highest concentration that has been reported in this detergent is about 0.5 mg/mL, and beyond this precipitates can be observed. This behaviour can be monitored with SEC [67]. For DDM-purified material, SEC purification was not suitable for completely removing the contaminating proteins [67], but a second affinity purification step can be applied such as FLAG affinity chromatography. However, the yield of FLAG affinity steps is variable and for CFTR it remains about 20–30 % because of low binding affinity. The compromise between yield and purity is commonly encountered in protein purification and is particularly difficult for membrane proteins.

Thus, CFTR behaved very differently depending on which detergent was used in the purification and which version of the protein was expressed—even though the purification methods were broadly similar. In the DDM purification buffer, 1 M salt and 20 % glycerol are used to maintain the quality of the protein [73, 74] but the solubility and monodispersity are still not sufficient for some experimental methods such as Cryo-EM and X-ray crystallography. High salt and glycerol are also problems for Cryo-EM [75] where the presence of these solutes greatly reduces the contrast between the protein particles and the background. DDM-purified CFTR also forms a range of oligomeric but non-identical complexes of up to 30 nm diameter. These appear to be in a dynamic equilibrium with monomeric particles because ultrafiltration with a 1 MDa cutoff device did not remove these larger complexes. Even when these larger aggregates were removed by SEC, they reappeared upon concentration and re-passaging down the SEC column. In conclusion, LPG gives a better solubility efficiency and prevents aggregation of purified full-length CFTR [67, 70, 76].

### Activity of purified CFTR and stability

The protein purified in LPG is highly mono-disperse as shown above, but has been reported to have less ATPase activity compared to the DDM-purified CFTR [67]. Isolated CFTR NBD1 loses its thermal unfolding transition detected by differential scanning calorimetry in the presence of LPG above a concentration of 0.05 % w/v [77]. Although this applies to the isolated domain, it does offer a plausible explanation for the loss of ATPase activity after LPG14 purification. However, from the thermal unfolding data, full-length CFTR purified in LPG shows a greater stability probably because the cytoplasmic NBDs are much more stable when associated with the other CFTR domains and where hydrophobic interfacial regions are buried (Cant, N. University of Manchester, PhD Thesis, 2013). Thus, it could be argued that stability measurements on isolated domains could be used to indicate trends in sensitivity to detergents and the effects of mutations on stability, but that studies on full-length membrane proteins which include the transmembrane domains are still essential.

CFTR and G551D CFTR ATPase activity can be compared after purification. The CFTR potentiator VX-770 can also applied in these studies because of its potentiation of CFTR G551D channel activity [78–82]. ATPase activity in CFTR preparations is inevitably susceptible to contaminating yeast membrane proteins having ATPase activity, especially the highly expressed plasma membrane H^+^-ATPase [83]. An ATPase activity inhibitor cocktail that includes Sch28080, sodium thiocyanate (SCN) and oligomycin inhibits F-, V- and P-type ATPases, but not CFTR channel gating [84]; hence, this cocktail can be employed to suppress the activity of the major yeast ATPases.

**Fig. 5:**
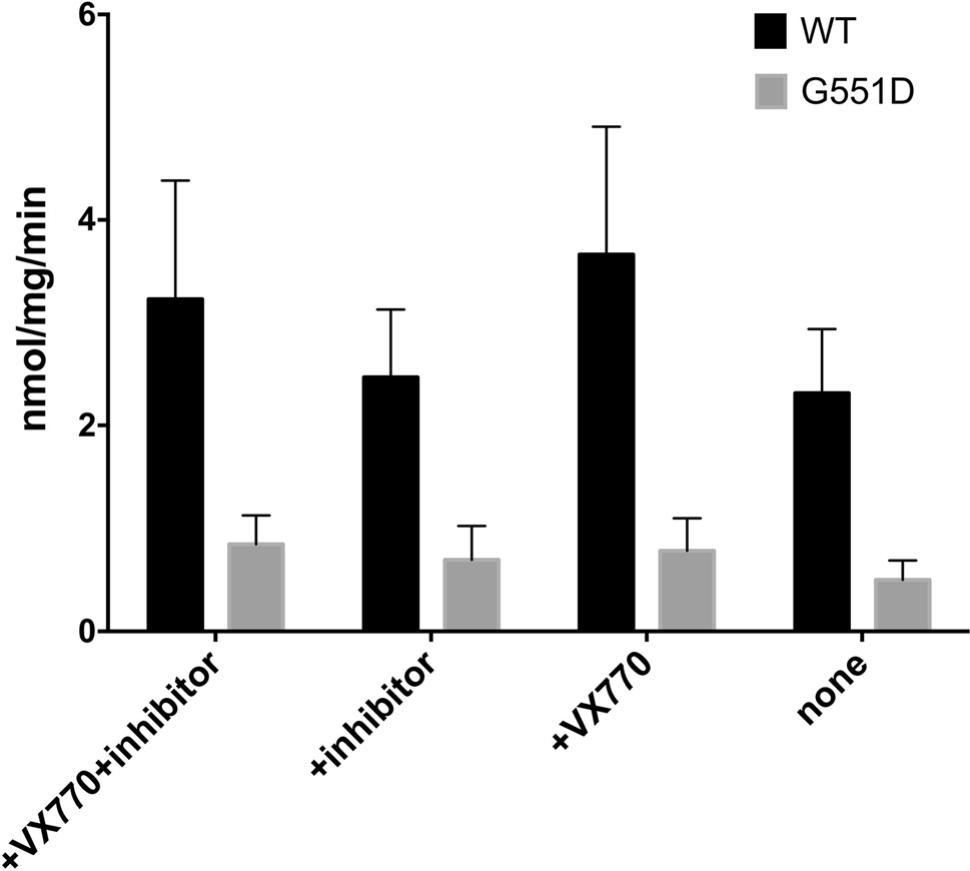
Comparison of the ATPase activity of WT and G551D CFTR with and without VX-770. Purified CFTR was reconstituted with lipids and its ATPase activity was measured using the Chifflet assay. The rate was measured at 25 °C at a concentration of 2 mM ATP. These experiments were repeated >3 times and *error bars* show the standard error of the mean. An inhibitor cocktail (+inhibitor) was also employed to check for contaminating ATPases of the V-, H- and P-type, as described earlier

As shown in Fig. 5, VX-770 does not have any effect on the ATPase activity of the purified and reconstituted G551D protein, which is consistent with the hypothesis that VX-770 may exert its effect through prolonging channel opening (which is associated with ATP binding rather than ATP hydrolysis) [6]. However, Eckford and co-workers [82] found PKA-phosphorylated G551D CFTR ATPase activity was stimulated by VX-770. They proposed that VX-770 potentiates defective channel gating in a phosphorylation-dependent manner whereas PKA phosphorylation did not seem to affect ATPase activity of purified chicken CFTR (Dr. Natasha Cant Ph.D thesis, University of Manchester 2013). Reconstitution has been shown to stimulate purified CFTR’s ATPase activity [67, 82]. Purified WT CFTR has much higher ATPase activity than G551D CFTR, as expected. These data are interesting and may be informative for considering the effects of detergent purification on activity; however, because the intrinsic ATPase activity of CFTR is relatively low ([85]—e.g. turnover of 0.1/s) the presence of even small amounts of highly active contaminating ATPases could be misleading and this urges caution in interpreting these data.

## Stability of CFTR as measured by the CPM assay

As discussed above, the CPM assay has been used for assessing the thermal stability of membrane proteins [63, 86–88]. The CPM (*N*-[4-(7-diethylamino-4-methyl-3-coumarinyl)phenyl]maleimide) dye is a thiol-specific fluorophore and it will fluoresce strongly in its thiol-linked state [89]. When incubated with protein, it will form a covalent bond with exposed cysteine residues [88]. Upon thermal denaturation, more cysteine residues will become surface exposed, resulting in the fluorescence yield increasing significantly. Only a small amount of protein is used in the CPM assay because the fluorescence read-out from the dye is very sensitive. Other advantages are that it is compatible with several detergents and exhibits a high signal-to-noise ratio [86–88]. The assay does require the presence of several Cys residues in a protein and (for measurement of thermal stability) that some of these Cys residues are secluded from the dye when the protein is in the folded state. Hence, it is less applicable to small proteins with few Cys residues. Nevertheless, it can, for example, be applied to measure the high-temperature unfolding of GFP which is a small protein, but one that fortuitously has 2 Cys residues—one buried and one surface exposed.

CPM, like many fluorescent dyes employed in biochemistry, is hydrophobic and photo-sensitive and, therefore, needs careful handling. It is recommended when making up CPM stock solutions in DMSO to store these solutions in glass vials at −20 °C and in the dark and then to dilute to the required experimental concentration just before use. Whenever possible, it is also advisable to use glass microsyringes for transfer and dilution of CPM stocks rather than the plastic tips employed routinely in biochemistry. The binding of CPM to proteins is affected by thiol-containing reducing agents such as DTT, so these must be removed from the protein and buffer samples before undertaking the assay. Reducing agent is normally present where protein aggregates are a problem, especially with higher protein concentrations (>1 mg/mL). However, the sensitivity of the CPM assay means that purified protein will not normally need to be concentrated, thus removal of DTT or reducing agent removal can be done just prior to assay. The concentration of membrane proteins used for the assay will typically be below 0.1 mg/mL at this stage. It is important to include a buffer control for all CPM-based assays. This is particularly important for membrane proteins where buffers will contain detergent in a micellar form. Although CPM is almost non-fluorescent in water, it does have a significant fluorescence when it is associated with detergent micelles.

### Application of the CPM assay to CFTR

**Fig. 6:**
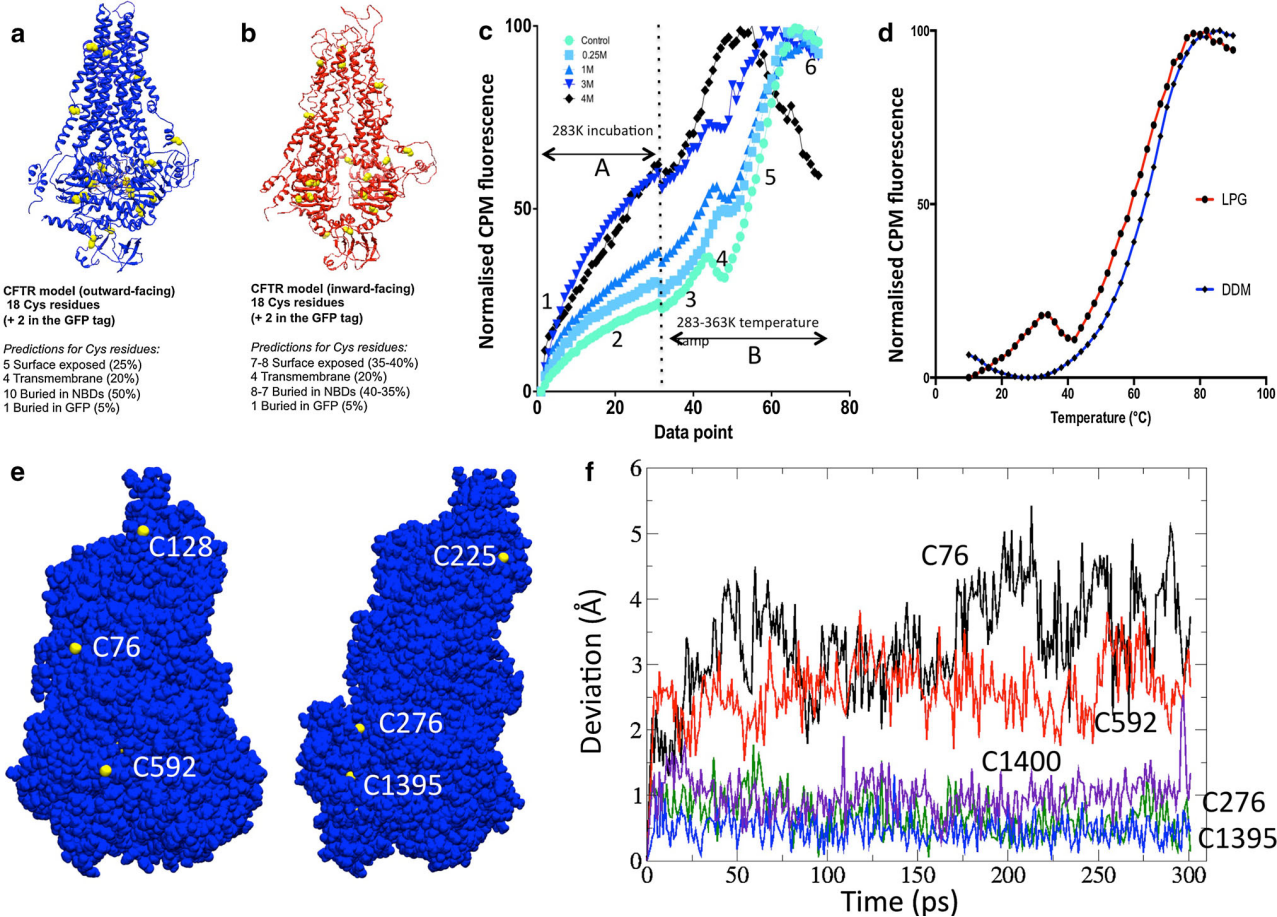
**a**, **b** Homology models for CFTR in the **a** outward-facing state (*blue ribbons*) and **b** inward-facing state (*red ribbons*). The locations of the Cys residues in each model are indicated by the *yellow* space-filling atoms, and predictions about their exposure to solvent are listed below each model. **c** The unfolding of CFTR by thermal or chemical denaturation as detected by CPM fluorescence changes. The *light green line* shows purified G551D CFTR in an LPG-containing buffer. *Other lines* show the same protein but in the presence of 0.25–4 M Guanidium HCl as indicated by the key, *top left*. The experiment is initiated by the injection of CPM dye into a protein-containing cuvette at 10 °C which is then monitored for 30 min (phase A). The sample is then heated to 90 °C (phase B). Various changes in the CPM fluorescence are indicated: *1* immediate fluorescence from CPM in buffer (unbound to protein). *2* Kinetics of CPM binding to solvent-exposed Cys residues. For protein chemically denatured (*black line*), nearly all Cys residues are initially exposed. *3* Rise in CPM fluorescence as thermal motion exposes more Cys residues to solvent and CPM. *4* Thermal quenching of CPM fluorescence as the sample is heated (protein is still predominantly folded for the *green line*). *5* Cooperative thermal unfolding of the entire protein. *6* Continued thermal quenching of CPM fluorescence after complete unfolding of the protein. Data point *scale* represents time (1 min/point—phase A) and then temperature (phase B—heating rate = 2 °C/min with 1 min/point). **d** The thermal denaturation phase for G551D CFTR purified in a non-ionic detergent (DDM, *blue line*) and an anionic lyso-lipid (LPG, *red line*). **e** Two views of the surface of a CFTR homology model based on the outward-facing Sav1866 structure, with the sulphur atoms of Cys residues coloured *yellow* whilst all other atoms are *blue*. The TMDs and NBDs are towards the *top* and *bottom*, respectively. C128 and C225 are in membrane-spanning portions and will probably be buried by surrounding lipid or detergent. **b** Deviations of the Cys sulphur atoms for five selected residues over the time course of a molecular dynamics simulation. The highly surface-exposed residues C76 (*black line*) and C592 (*red line*) show much greater mobility than the mostly buried residues C1395 (*blue*) and C276 (*green*). Hence, the former two residues are likely to be labelled by CPM before thermal denaturation. The completely buried residue C1400 (*indigo*) shows similar dynamics to C1395 and C276

There are a total of 18 Cys residues in the full-length WT human CFTR, 6 predicted to be in the TMDs, 11 in the NBDs and 1 in the R-region [90]. Examination of CFTR homology models [90] implies that 5 Cys are exposed in the outward-facing conformation and 7–8 Cys are exposed in the inward-facing state—see Fig. 6 [91]. In either conformation, there are still predicted to be a majority of Cys residues buried. At the beginning of the CPM assay, surface-exposed Cys will react with the CPM dye at the initial incubation temperature (in this case 10 °C to favour the native state). The buried Cys residues will probably only be exposed when the protein becomes unfolded. These predictions are based on models that are static snapshots of the CFTR structure, and CFTR may have more flexibility than expected due to the presence of the detergent. Nevertheless, it is possible to predict that CFTR will have at least 12 Cys that are partly buried and 6 surface-exposed Cys.

Figure 6 shows typical data for CFTR unfolding as measured using the CPM assay. In aqueous buffers, CPM fluorescence is low, but greatly increases upon formation of the covalent adduct with Cys residues in a protein. As shown in Fig. 6c, there is an increase in CPM fluorescence when CPM is added to purified CFTR protein due to adduct formation with the surface-exposed Cys residues (phase 2—Fig. 6c). The very fast initial increase in CPM fluorescence indicated by the number 1 is probably due to intrinsic CPM fluorescence in the buffer. Although the CPM fluorescence in aqueous buffers is normally extremely low, when detergent micelles are present, CPM can partition into the micelle and shows some significant fluorescence, as stated above. When CFTR is completely denatured by Guanidium HCl, the CPM fluorescence increases in phase ‘2’ to about 60 % of the total signal over a period of about 30 min at 10 °C (Fig. 6c, black line) with a half-time for the Cys adduct formation of about 10 min at this temperature. Presumably the increase does not reach 100 % because the membrane-spanning portions of the protein are relatively resistant to this water-soluble chaotrope. Subsequent heating of the Guanidium-denatured protein gives a further increase in CPM fluorescence—probably due to the exposure of Cys residues that are in the membrane-spanning portions of the protein. The final phase (phase 6) of the heating of the protein is characterised by a loss of the fluorescence yield due to thermal quenching. In contrast, folded CFTR (green line) gives only a small increase in CPM fluorescence at 10 °C (solid line, Fig. 6), equivalent to about 20 % of the total signal. This probably represents the small number of surface-exposed Cys residues in the folded CFTR protein—i.e. about 5 of the 20 available residues in the CFTR-GFP construct (Fig. 6a, b). A large increase in fluorescence is observed upon heating and thermal denaturation of the protein in the absence of Guanidium HCl as in this case, about 80 % of the Cys residues are buried. Hence, the CPM-derived fluorescence data are broadly consistent with the available homology models for CFTR and interestingly are more consistent with the outward-facing state rather than the inward-facing conformation. After complete thermal unfolding of the protein at approximately 78 °C, a decrease in CPM fluorescence yield is again observed due to thermal quenching. These experiments were performed using an Avacta Optim Fluorescence Spectrometer coupled to a Peltier element for heating, but a simple bench-top fluorescence spectrophotometer with an attached water bath for heating can also be employed.

**Fig. 7:**
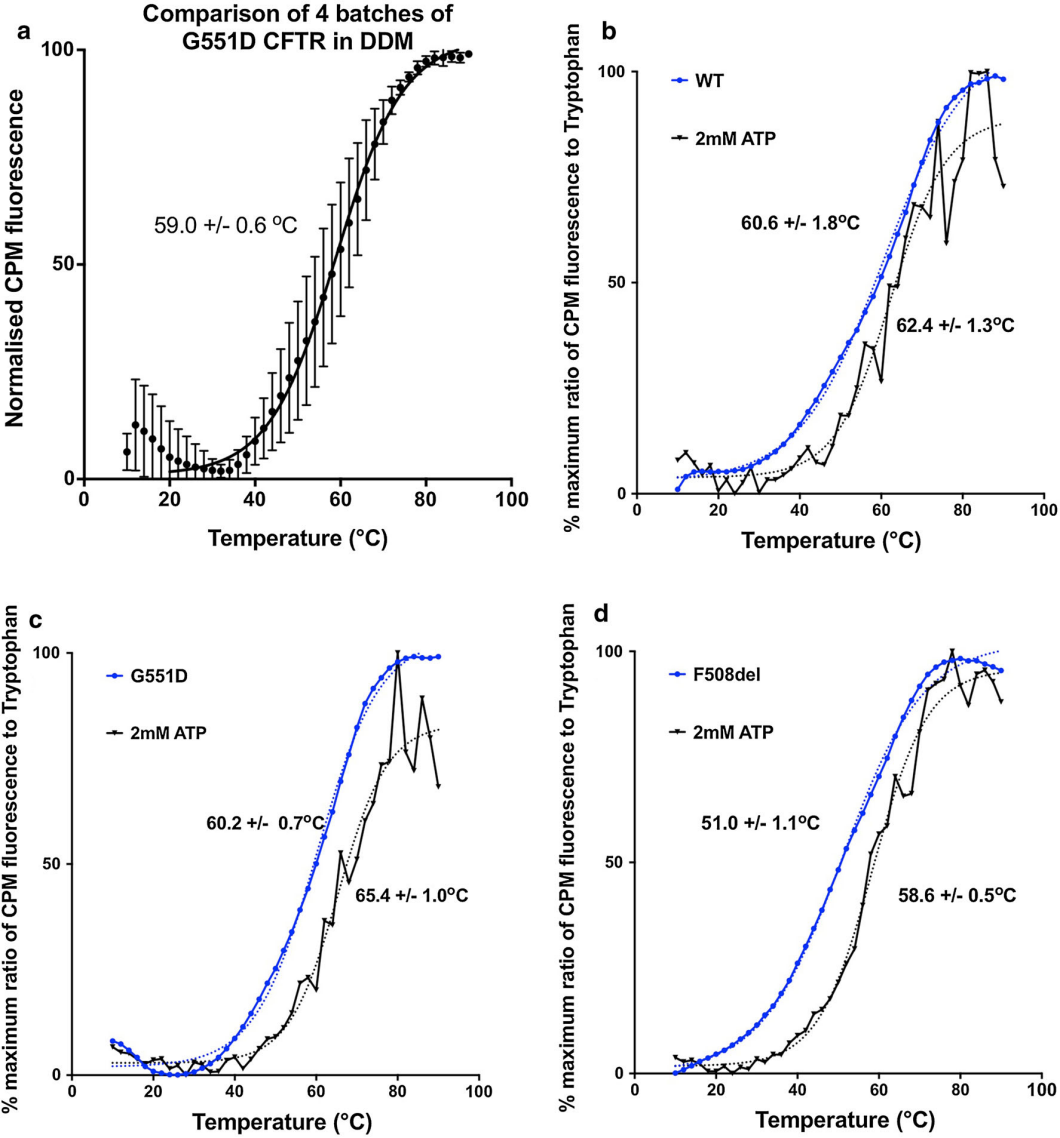
**a** Batch-to-batch variability in the thermal denaturation phase of four batches of DDM-purified G551D CFTR, as detected by CPM fluorescence changes (each batch was measured three times). Samples were heated at 2 °C/min. **b**–**d** Effects of ATP on the thermal unfolding transition of WT (**b**), G551D (**c**) and F508del CFTR purified in DDM. The presence of ATP stabilises all three constructs. The increased noise in the data recorded in the presence of ATP is due to the absorption of excitation light by ATP. F508del CFTR is globally destabilised versus WT and G551D CFTR by about 8 °C. The midpoint temperatures for the thermal unfolding transition are shown for each *curve* and the error for its estimation by the *curve* fitting is indicated

As the CPM fluorescence at any given temperature is a convolution of the accessibility/dynamics of available Cys residues, the kinetics of protein unfolding and CPM–Cys bond formation (temperature dependent) and the heating rate, this assay will give differing mid-point temperatures for protein unfolding depending on these various parameters. Hence, mid-point temperatures estimated for proteins will be somewhat unreliable and, because of hysteresis, will likely overestimate the stability of the protein at high heating rates. Conversely, slow heating rates will tend to underestimate the mid-point unfolding temperature because the time dependence of stability at a given moderate temperature will have a stronger influence. Hence, the assay can be used for comparative purposes as shown in Figs. 6 and 7, where the experimental parameters such as heating rate are the same. These data provide evidence that CFTR material purified in the anionic lyso-lipid LPG and in the non-ionic detergent DDM have a folded, cooperative tertiary structure. There are, however, differences in the unfolding profiles of LPG-purified and DDM-purified CFTR. Whilst DDM-purified CFTR unfolds in a single cooperative transition, LPG-purified CFTR shows a two-phase unfolding profile (Fig. 6d). The latter is characterised by an initial increase in CPM fluorescence between 10 and 35 °C, followed by a brief thermal quenching between 35 and 41 °C. This low-temperature and low-amplitude transition may be due to a localised unfolding of a sub-domain of CFTR in LPG, or may be due to increased dynamics of partly buried Cys residues. The main unfolding transitions for CFTR purified in either detergent are very similar, with mid-point temperatures of about 60–65 °C (Fig. 6d).

#### Molecular dynamics and modelling of Cys residues in CFTR

Of the 18 Cys residues in CFTR, and the 2 Cys residues in the GFP construct, some are clearly surface exposed in both inward- and outward-facing conformational models (Fig. 6a, b). In particular, Cys 832 (R-region) and Cys 1458 (C-term) are likely to be in disordered regions of the protein whilst Cys 48 in the GFP is likely to be surface exposed. Of the remaining Cys residues, Cys 78 and 647 are very close to regions of the CFTR structure that are known to be folded, hence may be at least partially buried. Residues 128, 225, 343, 866 are all in the membrane-spanning portions of the protein, and hence are likely to be secluded from CPM by detergent or lipid until the transmembrane domains are denatured. Most of the remaining Cys residues in CFTR are buried within the NBDs, although three display some exposure to the aqueous milieu in the outward-facing model (Fig. 6e). These are Cys residues at positions 276, 592 and 1395, with C592 and C1395 more buried than C276. Molecular dynamics of these Cys residues in a homology model for CFTR support the visual impression that C592 and C1395 are buried compared to C276 (Fig. 6f). These residues display dynamics that are similar to the completely buried C1400, whereas C276 shows much greater dynamics, similar to that modelled for C78. Hence, molecular dynamics simulation predicts that at least 4 Cys residues will be sufficiently surface-exposed to be labelled by CPM in the compact outward-facing configuration, viz., C276, C832, C1458 and C48 in the GFP tag. In addition, C78 and C647 may be labelled, but these residues are in regions of the CFTR structure for which little current structural data are available. Labelling of 4 of the 20 Cys residues in the purified CFTR-GFP construct with CPM is consistent with the fluorescence measurements shown in Fig. 6c.

### Comparison of CFTR mutations

Thermal unfolding transitions for various purified CFTR constructs as detected with the CPM assay are shown in Fig. 7. F508del CFTR has a broader thermal unfolding transition, whilst G551D CFTR shows a more cooperative unfolding that was similar to WT CFTR. G551D may be more stable at the beginning of the thermal denaturation transition, i.e. in the temperature range that is relevant physiologically. This may relate to the fact that the G551D mutation fixes the CFTR channel in a closed state, which may correlate with a more stable structural state for the whole protein. A re-scan of the CPM fluorescence for the thermally denatured protein is routinely monitored and should give a featureless profile with a negative slope, due to the thermal quenching of the CPM fluorescence. This also confirms that thermal unfolding is an irreversible process.

### Batch-to-batch variability and studies of stability in the presence of small molecules

The effects of the purification procedure may also have a major influence on the measured stability of a given membrane protein. Clearly, variability in purification conditions can result in a protein batch that contains significant amounts of already unfolded protein. An assessment of batch-to-batch variability in the measured stability is, therefore, important, particularly if results on the effects of mutations or small molecules on stability are desired. Figure 7 a illustrates the variability in the unfolding data across 4 different batches of G551D CFTR protein purified in the presence of DDM. The error bars indicate that there will be significant batch-to-batch variability, even for this well-behaved version of the protein. Hence, studies on the effects of small molecules on stability should be performed on the same batch of protein, and comparisons of the stability of CFTR mutants should be carried out on several batches of the protein before conclusions are drawn. Figure 7b–d shows the effects of ATP addition which gives a significant thermostabilisation of the purified full-length proteins, as might have been predicted given similar conclusions for the isolated nucleotide-binding domains [92]. In this latter study [92], the isolated WT and F508del NBD1 were both thermostabilised by about 10–15 °C by the presence of millimolar levels of ATP. For the full-length protein, the thermostabilisation of the protein is less, as shown in Fig. 7. For F508del CFTR, ATP-induced stabilisation is greatest, by about 8–10 °C in the midpoint transition, though at lower temperatures, the difference in the CPM-detected thermal unfolding is more noticeable. This may be taken as evidence that NBD unfolding may occur at lower temperatures. For WT and G551D CFTR, the degree of ATP-mediated themostabilisation is smaller, perhaps indicating that there is an upper limit for stabilisation via ATP binding to the NBDs.

## Summary

CFTR is a typical membrane protein that is somewhat difficult to express and purify. Stability is a major factor in the purification and structural analysis of membrane proteins, and CFTR is a good example of how increased stability can correlate with improved yield, purity and monodispersity. However, mutations that stabilise a given membrane protein may not always be associated with better activity. For example, the G551D mutation in CFTR improves its properties for structural studies, but by locking the protein into a channel closed conformation, it blocks its activity. Hence, it must be acknowledged that for some, perhaps the majority, of membrane proteins an inherent instability or flexibility may be crucial for the function of the protein. Structures of hyper-stabilised versions of membrane proteins must, therefore, be treated with some caution as they may bear less relevance for physiology. Indeed, for CFTR, even though the F508del version of the protein is by far the least stable, it is also probably the most relevant in terms of the disease, since loss of stability of F508del CFTR may be a major factor in its inability to reach the plasma membrane of the cell.

## References

[1] Mikhailov MV, Campbell JD, de Wet H (2005). 3-D structural and functional characterization of the purified KATP channel complex Kir6.2-SUR1. EMBO J.

[2] Bryan J, Aguilar-Bryan L (1999). Sulfonylurea receptors: ABC transporters that regulate ATP-sensitive K(+) channels. Biochim Biophys Acta.

[3] Bozoky Z, Krzeminski M, Muhandiram R (2013). Regulatory R region of the CFTR chloride channel is a dynamic integrator of phospho-dependent intra- and intermolecular interactions. Proc Natl Acad Sci USA.

[4] Ostedgaard LS, Baldursson O, Welsh MJ (2001). Regulation of the cystic fibrosis transmembrane conductance regulator Cl-channel by its R domain. J Biol Chem.

[5] Wang Y, Wrennal J, Cai Z (2014). Understanding how cystic fibrosis mutations disrupt CFTR function: from single molecules to animal models. Int J Biochem Cell Biol.

[6] Vergani P, Lockless SW, Nairn AC (2005). CFTR channel opening by ATP-driven tight dimerization of its nucleotide-binding domains. Nature.

[7] Bozoky Z, Krzeminski M, Muhandiram R (2013). Regulatory R region of the CFTR chloride channel is a dynamic integrator of phospho-dependent intra- and intermolecular interactions. Proc Natl Acad Sci USA.

[8] Ratjen F, Döring G (2003). Cystic fibrosis. Lancet.

[9] Goodman B, Percy W (2005). CFTR in cystic fibrosis and cholera: from membrane transport to clinical practice. AJP Adv Physiol Educ.

[10] Van Goor F, Hadida S, Grootenhuis PDJ (2011). Correction of the F508del-CFTR protein processing defect in vitro by the investigational drug VX-809. Proc Natl Acad Sci USA.

[11] Molinski S, Eckford PD, Pasyk S (2012). Functional rescue of F508del-CFTR using small molecule correctors. Front Pharmacol.

[12] He LH, Aleksandrov AA, An JL (2015). Restoration of NBD1 thermal stability is necessary and sufficient to correct delta F508 CFTR folding and assembly. J Mol Biol.

[13] Farinha CM, Matos P (2016). Repairing the basic defect in cystic fibrosis—one approach is not enough. FEBS J.

[14] Riordan JR (2008). CFTR function and prospects for therapy. Annu Rev Biochem.

[15] Serohijos AW, Hegedus T, Aleksandrov AA (2008). Phenylalanine-508 mediates a cytoplasmic-membrane domain contact in the CFTR 3D structure crucial to assembly and channel function. Proc Natl Acad Sci USA.

[16] He L, Kota P, Aleksandrov AA (2013). Correctors of DeltaF508 CFTR restore global conformational maturation without thermally stabilizing the mutant protein. FASEB J Off Publ Fed Am Soc Exp Biol.

[17] He L, Aleksandrov AA, Serohijos AW (2008). Multiple membrane-cytoplasmic domain contacts in the cystic fibrosis transmembrane conductance regulator (CFTR) mediate regulation of channel gating. J Biol Chem.

[18] Ward CL, Omura S, Kopito RR (1995). Degradation of CFTR by the ubiquitin-proteasome pathway. Cell.

[19] Kopito RR (1999). Biosynthesis and degradation of CFTR. Physiol Rev.

[20] Grove DE, Rosser MFN, Watkins RL (2011). Analysis of CFTR folding and degradation in transiently transfected cells. Cys Fibros Diagn Protoc Vol I Approaches Study Correct CFTR Defects.

[21] Cui L, Aleksandrov L, Chang XB (2007). Domain interdependence in the biosynthetic assembly of CFTR. J Mol Biol.

[22] Dalemans W, Barbry P, Champigny G (1991). Altered chloride ion channel kinetics associated with the delta F508 cystic fibrosis mutation. Nature.

[23] Denning GM, Anderson MP, Amara JF (1992). Processing of mutant cystic fibrosis transmembrane conductance regulator is temperature-sensitive. Nature.

[24] Sato S, Ward CL, Krouse ME (1996). Glycerol reverses the misfolding phenotype of the most common cystic fibrosis mutation. J Biol Chem.

[25] Pedemonte N, Galietta LJ (2012). Pharmacological correctors of mutant CFTR mistrafficking. Front Pharmacol.

[26] Eckford PD, Bear CE (2011). Targeting the regulation of CFTR channels. Biochem J.

[27] Meacham GC, Patterson C, Zhang WY (2001). The Hsc70 co-chaperone CHIP targets immature CFTR for proteasomal degradation. Nat Cell Biol.

[28] Becq F, Mettey Y, Gray MA (1999). Development of substituted benzo[c]quinolizinium compounds as novel activators of the cystic fibrosis chloride channel. J Biol Chem.

[29] Dormer RL, Dérand R, McNeilly CM (2001). Correction of delF508-CFTR activity with benzo(c)quinolizinium compounds through facilitation of its processing in cystic fibrosis airway cells. J Cell Sci.

[30] Pedemonte N, Lukacs GL, Du K (2005). Small-molecule correctors of defective deltaF508-CFTR cellular processing identified by high-throughput screening. J Clin Investig.

[31] Hutt DM, Herman D, Rodrigues AP (2009). Reduced histone deacetylase 7 activity restores function to misfolded CFTR in cystic fibrosis. Nat Chem Biol.

[32] Pedemonte N, Tomati V, Sondo E (2011). Dual activity of aminoarylthiazoles on the trafficking and gating defects of the cystic fibrosis transmembrane conductance regulator chloride channel caused by cystic fibrosis mutations. J Biol Chem.

[33] Van Goor F, Straley KS, Cao D (2006). Rescue of deltaF508-CFTR trafficking and gating in human cystic fibrosis airway primary cultures by small molecules. Am J Physiol Lung Cell Mol Physiol.

[34] Van Goor F, Hadida S, Grootenhuis PD (2011). Correction of the F508del-CFTR protein processing defect in vitro by the investigational drug VX-809. Proc Natl Acad Sci USA.

[35] Haq IJ, Gray MA, Garnett JP (2015). Airway surface liquid homeostasis in cystic fibrosis: pathophysiology and therapeutic targets. Thorax.

[36] Clancy JP, Rowe SM, Accurso FJ (2012). Results of a phase IIa study of VX-809, an investigational CFTR corrector compound, in subjects with cystic fibrosis homozygous for the F508del-CFTR mutation. Thorax.

[37] Farinha CM, Sousa M, Canato S (2015). Increased efficacy of VX-809 in different cellular systems results from an early stabilization effect of F508del-CFTR. Pharmacol Res Perspect.

[38] Sampson HM, Robert R, Liao J (2011). Identification of a NBD1-binding pharmacological chaperone that corrects the trafficking defect of F508del-CFTR. Chem Biol.

[39] Veit G, Avramescu RG, Perdomo D (2014). Some gating potentiators, including VX-770, diminish deltaF508-CFTR functional expression. Sci Transl Med.

[40] Cholon DM, Quinney NL, Fulcher ML (2014). Potentiator ivacaftor abrogates pharmacological correction of deltaF508 CFTR in cystic fibrosis. Sci Transl Med.

[41] Osmanbeyoglu HU, Wehner JA, Carbonell JG (2010). Active machine learning for transmembrane helix prediction. BMC Bioinform.

[42] Zhang Z, Kuipers G, Niemiec L (2015). High-level production of membrane proteins in *E. coli* BL21(DE3) by omitting the inducer IPTG. Microb Cell Factories.

[43] Whittaker MM, Whittaker JW (2014). Expression and purification of recombinant *Saccharomyces cerevisiae* mitochondrial carrier protein YGR257Cp (Mtm1p). Protein Expr Purif.

[44] Lundstrom K, Wagner R, Reinhart C (2006). Structural genomics on membrane proteins: comparison of more than 100 GPCRs in 3 expression systems. J Struct Funct Genomics.

[45] Garavito RM, Picot D, Loll PJ (1996). Strategies for crystallizing membrane proteins. J Bioenerg Biomembr.

[46] Seddon AM, Curnow P, Booth PJ (2004). Membrane proteins, lipids and detergents: not just a soap opera. Biochim Biophys Acta.

[47] Linke D (2009). Detergents: an overview. Methods Enzymol.

[48] Krueger-Koplin RD, Sorgen PL, Krueger-Koplin ST (2004). An evaluation of detergents for NMR structural studies of membrane proteins. J Biomol NMR.

[49] Fethiere J (2007). Three-dimensional crystallization of membrane proteins. Methods Mol Biol.

[50] Kim JM, Wu SP, Tomasiak TM (2015). Subnanometre-resolution electron cryomicroscopy structure of a heterodimeric ABC exporter. Nature.

[51] Pollock NL, Satriano L, Zegarra-Moran O (2016). Structure of wild type and mutant F508del CFTR. A small-angle X-ray scattering study of the protein-detergent complexes. J Struct Biol.

[52] Le Brun AP, Clifton LA, Holt SA (2016). Deuterium labeling strategies for creating contrast in structure-function studies of model bacterial outer membranes using neutron reflectometry. Methods Enzymol.

[53] Yang ZR, Wang C, Zhou QX (2014). Membrane protein stability can be compromised by detergent interactions with the extramembranous soluble domains. Protein Sci.

[54] Aleksandrov LA, Jensen TJ, Cui LY (2015). Thermal stability of purified and reconstituted CFTR in a locked open channel conformation. Protein Expr Purif.

[55] Geertsma ER, Groeneveld M, Slotboom DJ (2008). Quality control of overexpressed membrane proteins. Proc Natl Acad Sci USA.

[56] Hattori M, Hibbs RE, Gouaux E (2012). A fluorescence-detection size-exclusion chromatography-based thermostability assay for membrane protein precrystallization screening. Structure.

[57] Oldham ML, Hite RK, Steffen AM (2016). A mechanism of viral immune evasion revealed by cryo-EM analysis of the TAP transporter. Nature.

[58] Bai XC, Yan CY, Yang GH (2015). An atomic structure of human gamma-secretase. Nature.

[59] Nogales E, Scheres SHW (2015). Cryo-EM: a unique tool for the visualization of macromolecular complexity. Mol Cell.

[60] Zhou HX, Cross TA (2013). Influences of membrane mimetic environments on membrane protein structures. Annu Rev Biophys.

[61] Tate CG (2012). A crystal clear solution for determining G-protein-coupled receptor structures. Trends Biochem Sci.

[62] Moon CP, Fleming KG (2011). Using tryptophan fluorescence to measure the stability of membrane proteins folded in liposomes. Methods Enzymol.

[63] Kohlstaedt M, von der Hocht I, Hilbers F (2015). Development of a Thermofluor assay for stability determination of membrane proteins using the Na(+)/H(+) antiporter NhaA and cytochrome c oxidase. Acta Crystallogr D Biol Crystallogr.

[64] Huang P, Liu Q, Scarborough GA (1998). Lysophosphatidylglycerol: a novel effective detergent for solubilizing and purifying the cystic fibrosis transmembrane conductance regulator. Anal Biochem.

[65] Huang P, Stroffekova K, Cuppoletti J (1996). Functional expression of the cystic fibrosis transmembrane conductance regulator in yeast. Biochim Biophys Acta.

[66] O’ryan L, Rimington T, Cant N et al (2012) Expression and purification of the cystic fibrosis transmembrane conductance regulator protein in *Saccharomyces cerevisiae*. Jove J Vis Exp 61:e3860 10.3791/3860 PMC3460588 22433465

[67] Pollock N, Cant N, Rimington T et al (2014) Purification of the cystic fibrosis transmembrane conductance regulator protein expressed in *Saccharomyces cerevisiae*. J Vis Exp (87):51447. doi:10.3791/51447 10.3791/51447 PMC4181556 24893839

[68] Yang Z, Wang C, Zhou Q (2014). Membrane protein stability can be compromised by detergent interactions with the extramembranous soluble domains. Protein Sci Publ Protein Soc.

[69] Zhang L, Aleksandrov LA, Zhao Z (2009). Architecture of the cystic fibrosis transmembrane conductance regulator protein and structural changes associated with phosphorylation and nucleotide binding. J Struct Biol.

[70] Huang P, Liu Q, Scarborough GA (1998). Lysophosphatidylglycerol: a novel effective detergent for solubilizing and purifying the cystic fibrosis transmembrane conductance regulator. Anal Biochem.

[71] Newstead S, Kim H, von Heijne G (2007). High-throughput fluorescent-based optimization of eukaryotic membrane protein overexpression and purification in Saccharomyces cerevisiae. Proc Natl Acad Sci USA.

[72] Newstead S, Ferrandon S, Iwata S (2008). Rationalizing alpha-helical membrane protein crystallization. Protein Sci.

[73] Vogel R, Fan GB, Sheves M (2001). Salt dependence of the formation and stability of the signaling state in G protein-coupled receptors: evidence for the involvement of the Hofmeister effect. Biochemistry.

[74] Gekko K, Timasheff SN (1981). Thermodynamic and kinetic examination of protein stabilization by glycerol. Biochemistry.

[75] Rubinstein JL (2007). Structural analysis of membrane protein complexes by single particle electron microscopy. Methods.

[76] Ramjeesingh M, Li C, Garami E (1997). A novel procedure for the efficient purification of the cystic fibrosis transmembrane conductance regulator (CFTR). Biochem J.

[77] Yang Z, Wang C, Zhou Q (2014). Membrane protein stability can be compromised by detergent interactions with the extramembranous soluble domains. Protein Sci.

[78] Accurso FJ, Rowe SM, Clancy JP (2010). Effect of VX-770 in persons with cystic fibrosis and the G551D-CFTR mutation. N Engl J Med.

[79] Ramsey BW, Davies J, McElvaney NG (2011). A CFTR potentiator in patients with cystic fibrosis and the G551D mutation. N Engl J Med.

[80] Van Goor F, Hadida S, Grootenhuis PD (2009). Rescue of CF airway epithelial cell function in vitro by a CFTR potentiator, VX-770. Proc Natl Acad Sci USA.

[81] Yu H, Burton B, Huang CJ (2012). Ivacaftor potentiation of multiple CFTR channels with gating mutations. J Cyst Fibros.

[82] Eckford PD, Li C, Ramjeesingh M (2012). Cystic fibrosis transmembrane conductance regulator (CFTR) potentiator VX-770 (ivacaftor) opens the defective channel gate of mutant CFTR in a phosphorylation-dependent but ATP-independent manner. J Biol Chem.

[83] Carmelo V, Bogaerts P, Sa-Correia I (1996). Activity of plasma membrane H+-ATPase and expression of PMA1 and PMA2 genes in *Saccharomyces cerevisiae* cells grown at optimal and low pH. Arch Microbiol.

[84] Schultz BD, Bridges RJ, Frizzell RA (1996). Lack of conventional ATPase properties in CFTR chloride channel gating. J Membr Biol.

[85] Al-Zahrani A, Cant N, Kargas V, Rimington T, Aleksandrov L, Riordan JR, Ford RC (2015). Structure of the cystic fibrosis transmembrane conductance regulator in the inward-facing conformation revealed by single particle electron microscopy. AIMS Biophys.

[86] Lee SC, Bennett BC, Hong WX (2013). Steroid-based facial amphiphiles for stabilization and crystallization of membrane proteins. Proc Natl Acad Sci USA.

[87] Sonoda Y, Newstead S, Hu NJ (2011). Benchmarking membrane protein detergent stability for improving throughput of high-resolution X-ray structures. Structure.

[88] Alexandrov AI, Mileni M, Chien EY (2008). Microscale fluorescent thermal stability assay for membrane proteins. Structure.

[89] Ayers FC, Warner GL, Smith KL (1986). Fluorometric quantitation of cellular and nonprotein thiols. Anal Biochem.

[90] Mornon JP, Lehn P, Callebaut I (2008). Atomic model of human cystic fibrosis transmembrane conductance regulator: membrane-spanning domains and coupling interfaces. Cell Mol Life Sci.

[91] Mornon JP, Lehn P, Callebaut I (2009). Molecular models of the open and closed states of the whole human CFTR protein. Cell Mol Life Sci CMLS.

[92] Protasevich I, Yang Z, Wang C (2010). Thermal unfolding studies show the disease causing F508del mutation in CFTR thermodynamically destabilizes nucleotide-binding domain 1. Protein Sci Publ Protein Soc.

